# Interpersonal Emotion Regulation: Consequences for Brands in Customer Service Interactions

**DOI:** 10.3389/fpsyg.2022.872670

**Published:** 2022-06-09

**Authors:** Crystal Reeck, N. Nur Yazgan Onuklu

**Affiliations:** ^1^Marketing - Fox School of Business, Temple University, Philadelphia, PA, United States; ^2^Department of Business Administration, TED University, Ankara, Turkey

**Keywords:** interpersonal emotion regulation (IER), brand loyalty (BL), brand trust (BT), emotions, customer service

## Abstract

This research demonstrates that interpersonal emotion regulation—attempts to manage others’ feelings—influences consumer perceptions during sales and service interactions impacting brand trust and loyalty. Building on previous research linking interpersonal emotion regulation to improved outcomes between people, across five experiments, we demonstrate that antecedent-focused interpersonal emotion regulation strategies result in enhanced brand loyalty and brand trust compared to response-focused interpersonal emotion regulation strategies. Analysis of mediation models reveals this effect is explained by changes in the consumer’s emotions, which in turn influence evaluations of the service interaction and subsequently impacts brand outcomes. We identify reactance as a moderator of this effect, such that customers with low (high) reactance to interpersonal regulation attempts exhibit more (less) favorable brand trust and loyalty evaluations. Further, we demonstrate that the visibility of interpersonal emotion regulation represents an important boundary condition. These findings support the process model of interpersonal emotion regulation and generate important insights for both theory and practice.

## Introduction

Imagine the frustration a consumer feels when a new laptop suddenly stops working, or the anxiety new parents feel shopping for their first car seat. Salespeople or customer service employees frequently interact with customers experiencing negative emotions for a variety of reasons (Grandey et al., 2004). In these interactions, the behaviors of frontline service employees are the key determinant of customer evaluations of the service (Hartline et al., 2000). The 2011 Customer Experience Impact Report (Oracle, 2011) reveals that 89% of consumers began doing business with a competitor following a poor customer experience and 73% of consumers state that the behavior of employees or customer service representatives is important to creating a memorable experience that causes them to remain loyal to a brand. The risks of failing to adequately manage customer emotions were apparent when United Airlines had a severe brand crisis in 2017, causing its stock to drop $1.4 billion (Lucinda, 2017) after a crying passenger was forcibly removed from a United Airlines plane.

The present research examines how frontline employees’ efforts to control customers’ emotions—interpersonal emotion regulation (IER)—impact customers’ evaluations of the service interaction, brand trust, and loyalty. Emotion regulation is a goal-oriented process influencing the type, timing, experience, and expression of emotions (Gross, 1998b). Emotion regulation strategies which target earlier psychological stages in the emotion generation process (antecedent-focused) produce more adaptive outcomes than strategies which target later psychological stages (response-focused) in the emotion generation process (Gross, 1998a). Research in emotion regulation has largely focused on examining how people manage their own emotions. However, some work has examined IER—how people regulate others’ emotions (Reeck et al., 2016). IER takes place in a variety of contexts (Niven et al., 2009), including between romantic partners (Vangelisti et al., 1991; Bloch et al., 2014; Levenson et al., 2014), medical doctors and their patients (Francis et al., 1999), parents and children (Kopp, 1989; Díaz et al., 1990; Spinrad et al., 2004), coworkers (Rafaeli and Sutton, 1989; Lively, 2000; Niven et al., 2012), and employees and customers (Little et al., 2013). IER typically results in improved interpersonal outcomes, including greater marital satisfaction (Bloch et al., 2014) and enhanced trust (Niven et al., 2012; Williams, 2007).

The present research examines whether similar benefits of IER might emerge between customers and brands, such that customers who are the target of antecedent-focused IER may experience more brand loyalty and brand trust. Across five studies we investigate the impact of IER on consumer emotions, service evaluations, and brand perceptions, and identify factors that can improve customer-brand relationships. We begin with an overview of the literature on customer relationships to brands and emotion regulation. We then present evidence from a pilot study based on a survey of over 40,000 patients of the United Kingdom’s National Health Service demonstrating that provision of emotional support by hospital staff is positively correlated with patients’ evaluations of the care they received and their trust in the hospital. Next, we present five experiments that tested IER strategies in different sales and service contexts. Our investigations reveal that antecedent-focused emotion regulation improves customers’ subjective emotions and results in higher brand loyalty and brand trust than response-focused IER and having no IER at all. The influence of IER on brand outcomes is mediated by customer emotional responses, which in turn alter perceptions of the service interaction. Moreover, we show that IER influences brand outcomes irrespective of the emotion-eliciting event, such that benefits to brand trust and loyalty are observed not only when the customer’s emotion arises from an issue with the product or service but also when the customer’s emotion arises for reasons unrelated to the brand. Reactance also moderates this effect, presenting an important boundary condition for the benefits to brands of IER. We also demonstrate that enhancing the visibility of the attempted IER removes the negative influence of response-focused emotion regulation by increasing perceptions of the benevolence of the employee’s actions. Our research demonstrates that IER not only benefits consumer perceptions of the interaction with the customer service representative but can also positively impact brand perceptions and loyalty.

The present research addresses an important research gap: clarifying how the strategies used to manage customers’ emotions impact the customers’ brand attitudes. The current research makes four substantive contributions. First, it builds on the process model of emotion regulation (Gross, 1998b,Gross, 2015), which has previously demonstrated that antecedent-focused emotion regulation results in improved outcomes compared to response-focused emotion regulation when people are attempting to regulate their own feelings. Here, we demonstrate similar benefits of antecedent-focused emotion regulation when it is applied interpersonally. Second, it extends prior research in emotional labor, which focuses on the consequences of the strategies employees use to manage their own emotions, to instead examine the consequences of the strategies employees use to manage customers’ emotions. Third, the current research builds on prior demonstrations that IER can improve relationships between people by showing that these benefits can also emerge between customers and brands. Finally, these findings build upon and complement theoretical frameworks that emphasize the role of emotions in fostering consumers’ connections with brands (Jacoby and Chestnut, 1978; Fournier, 1998; Albert and Merunka, 2013). These findings extend understanding regarding the ramifications of IER to encompass brand outcomes and generate practical insights for brand management and customer service.

### Conceptual Background

#### Brand Relationships and Customer Emotions

Customers have relationships with brands, and the quality of those relationships has numerous influences on key outcomes (Fournier, 1998). Two key aspects of customer-brand relationships are brand loyalty and brand trust. Brand loyalty is defined as a commitment to repurchase the same product or service consistently in the future despite possible influences having the potential to create switching behavior (Oliver, 1999). Brand loyalty results in higher willingness to pay for the brand as consumers perceive a unique value of the brand that other brands lack (Jacoby and Chestnut, 1978; Reichheld and Teal, 1996). Brand trust is a consumer’s confidence in a brand’s reliability and integrity (Morgan and Hunt, 1994). Brand trust contributes to the development of brand loyalty (Chaudhuri and Holbrook, 2001) and brand loyalty is associated with higher market share, cash flows, and profits (Chaudhuri and Holbrook, 2001; Homburg et al., 2009; Morgan and Rego, 2009; Watson et al., 2015).

Importantly, brand loyalty arises because of customers’ emotional attachment and affective commitment to the brand (Jacoby and Chestnut, 1978; Dick and Basu, 1994; Oliver, 1999; Thomson et al., 2005; Grisaffe and Nguyen, 2011; Iglesias et al., 2011). Indeed, affective commitment to the brand has been shown to be a stronger predictor of brand loyalty than economic considerations (Evanschitzky et al., 2006), highlighting the importance of customer emotions in forging brand relationships.

Failures by brands can disrupt their relationship with their customers. Service and product failures can upset customers and damage their relationship with the brand (Smith et al., 1999; Aaker et al., 2004). Customers’ negative emotions in response to such failures, particularly their anger, predict negative outcomes for the brand, including decreased customer satisfaction, intentions to spread negative word-of-mouth, decreased repurchase intentions, and intentions to engage third-party action (Smith and Bolton, 2002; Bonifield and Cole, 2007; Kalamas et al., 2008). Thus, addressing and assuaging customers’ negative emotions to service failures is an important aspect of recovery from brand failures. Critically, the quality of the relationship between customers and brands can mitigate the negative consequences of brand failures (Berry, 1995; Tax et al., 1998; Hess et al., 2003; Aaker et al., 2004; Huber et al., 2010). For example, customers of brands that feature more personalization have greater tolerance for brand failures (Berry, 1995), and customers who view the brand as a quality partner following a transgression are more likely to preserve their relationship with the brand (Aaker et al., 2004). These findings suggest that steps brands take to improve their relationships with their customers can not only enhance the customers’ connection to the brand but also help insulate the brand from negative consequences in the wake of brand transgressions. Moreover, addressing customers’ negative emotions can also help limit the damage done by brand failures.

IER has been shown to improve relationships and increase trust between people (Niven et al., 2007, 2012; Williams, 2007; Bloch et al., 2014). Here, we examine whether similar effects might emerge between people and brands. For example, if brand representatives implement IER to address customers’ negative emotions following a service failure, the resulting improvement in customers’ emotional states may both mitigate negative consequences for the brand and improve customers’ brand loyalty and brand trust. In contrast to previous work examining how customers manage their own emotional reactions to brand failures (Strizhakova et al., 2012; [Bibr B100][Bibr B100]; Balaji et al., 2017), here we examine the influence of brand representatives’ attempts to manage customer emotional responses. We therefore next turn our attention to the literature on emotion regulation and the impact of different emotion regulation strategies.

#### Emotion Regulation

Emotion regulation is a goal-oriented process influencing the type, timing, experience and expression of emotions (Gross, 1998a). When a patient anxiously waiting to see a doctor reduces feelings of distress by reminding herself of the benefits of regular health check-ups, she is regulating her emotions. Similarly, a customer is regulating emotions when he avoids yelling at a salesperson when his return request is declined. The majority of research in this area has focused on how people regulate their own emotions. There are numerous examples of cases where emotions may be inappropriate or unwanted in a particular situation (Gross and Jazaieri, 2014), and emotion regulation enables individuals to control their emotional experience and expression. People can regulate their emotions either consciously or implicitly (Mauss et al., 2007; Gyurak et al., 2011).

Prior research has distinguished between broad categories of emotion regulation strategies: antecedent-focused and response-focused emotion regulation (Gross, 1998b). According to the modal model of emotion, emotions arise through iterative, sequential processes. Features of a given situation are attended to by the agent, who subsequently appraises and interprets their meaning which generates a specific emotional response and expression (Gross, 1998b,Gross, 1998a,^2015^). Antecedent-focused emotion regulation targets changing psychological processes arising early in the emotion generation process, before the emotional response is fully developed. Such strategies include changing the situation (Lazarus and Folkman, 1984), redirecting one’s attention (Rodriguez et al., 1989), or reinterpreting the meaning or impact of the emotional elicitor (Gross, 2001). In contrast, response-focused emotion regulation targets changing the emotional response that is generated by these antecedent psychological processes. Response-focused strategies include, for example, trying to maintain a poker face rather than expressing one’s strong inner emotional feelings (Gross, 1998a), smiling in order to make oneself feel better (Kleinke et al., 1998), or changing one’s breathing in order to relax (Thayer and Lane, 2000). These two classes of emotion regulation strategies typically have different affective consequences, with antecedent-focused emotion regulation typically resulting in larger changes in subjective feelings of emotion (Gross and Levenson, 1993; Gross, 1998a; Ray et al., 2010; Brans et al., 2013), reduced physiological responding associated with emotional events (Gross and Levenson, 1993; Gross, 1998a; Richards and Gross, 2000), enhanced memory encoding during emotional events (Richards and Gross, 2000; Richards et al., 2003; Hayes et al., 2010), and decreased neural activation in brain regions associated with emotional responding (Ochsner et al., 2004; Barrett et al., 2007; Goldin et al., 2008; Ochsner and Gross, 2008) compared with response-focused emotion regulation.

The strategies people use for managing their own emotions also have interpersonal consequences. People who habitually use antecedent-focused emotion regulation strategies typically have more close relationships and are better liked that those who habitually use response-focused emotion regulation strategies, and those who habitually use response-focused emotion regulation often experience less social support (Gross and John, 2003; Srivastava et al., 2009; English et al., 2012). For example, one longitudinal study found that students who reported greater habitual use of response-focused emotion regulation at the start of college had fewer close relationships at the end of college, while those who reported greater habitual use of antecedent-focused emotion regulation at the start of college had more close relationships and higher social status as measured by peer-reports at the end of college (English et al., 2012). Importantly, these effects of emotion regulation persisted even when controlling for baseline differences in social function and the Big Five personality traits. Laboratory studies have also revealed that response-focused emotion regulation strategies tend to disrupt interpersonal processes and relationship formation, producing increased blood pressure in conversation partners, reducing communication, impeding rapport, and diminishing memory for the social interaction (Butler et al., 2003; Richards et al., 2003). Training in antecedent-focused emotion regulation has also been shown to have positive influences on relationships and interpersonal processes. For example, training in antecedent-focused emotion regulation improved relationship satisfaction among married couples (Finkel et al., 2013) and enhanced support for conciliatory policies with Palestine among Israeli citizens (Halperin et al., 2013). Antecedent-focused emotion regulation therefore seems to result in better interpersonal outcomes than response-focused emotion regulation when people use it to manage their own feelings.

In the context of customer relationship management, past research has mostly focused on how the strategies employees use to manage their emotional expression influence employee well-being and performance. For many frontline employees, including customer service representatives, regulating one’s own emotions is part of their work responsibilities, termed “emotional labor” (Hochschild, 1991). Research examining the consequences of emotional labor has mainly explored outcomes of employees regulating how they express their own emotions to customers. In this context, antecedent-focused emotion regulation typically involves reappraising the interaction with the customer while response-focused emotion regulation typically involves suppressing the expression of negative emotions or faking positive emotions (Grandey, 2000, 2003; Brotheridge and Lee, 2003). In general, antecedent-focused emotion regulation tends to result in improved outcomes for employees’ personal well-being and performance compared to response-focused emotion regulation (Grandey and Gabriel, 2015). Whereas response-focused emotion regulation is associated with job burnout and reduced job satisfaction, antecedent-focused emotion regulation is associated with increased job satisfaction (Côté and Morgan, 2002; Judge et al., 2009; Hülsheger et al., 2010; Hülsheger and Schewe, 2011; Kammeyer-Mueller et al., 2013). Antecedent-focused emotion regulation also improves employee performance, resulting in more favorable evaluations and larger tips from customers compared to response-focused emotion regulation (Groth et al., 2009; Chi et al., 2011).

However, this literature has stopped short of examining how employees manage customer emotions and the consequences of such interpersonal emotion regulation strategies for the organizations they represent. Interacting with customers experiencing negative emotions is a regular occurrence for customer service representatives (Grandey et al., 2004) and how customers are treated emotionally in these instances is an important determinant of customer loyalty (Oracle, 2011). The present research addresses this gap by examining how interpersonal emotion regulation strategies shape customers’ emotional responses, their perceptions of the service interaction, and their brand loyalty and trust.

#### Interpersonal Emotion Regulation

Interpersonal emotion regulation involves attempting to change the nature, duration, timing, or intensity of the emotional experience and expression of another individual (Reeck et al., 2016). IER has been documented in wide-ranging contexts, including between romantic partners (Vangelisti et al., 1991; Bloch et al., 2014; Levenson et al., 2014), parents and children (Kopp, 1989; Díaz et al., 1990; Spinrad et al., 2004), coworkers (Rafaeli and Sutton, 1989; Lively, 2000; Niven et al., 2012), and employees and customers (Francis et al., 1999; Little et al., 2013). IER can result in positive benefits for both the person attempting regulation and the target of their efforts, including reduced negative and increased positive emotional experience (Kopp, 1989; Niven et al., 2011, 2012; Little et al., 2013) and improved ability to manage one’s own emotions (Kopp, 1989; Spinrad et al., 2004; Morris et al., 2015; Dore et al., 2017). Importantly, IER strategies can also be categorized as antecedent-focused, such as attempts to direct targets’ attention or reframe the meaning of emotional events, or response-focused, such as engaging in injunctive control by instructing people to change the behavioral expression of their emotions (Kopp, 1989; Ashforth and Kreiner, 2002; Little et al., 2013). The process model of IER (Reeck et al., 2016) posits that antecedent-focused IER should result in improved outcomes compared to response-focused IER. We therefore hypothesize that antecedent-focused IER will result in increased positive emotion and decreased negative emotion for customers compared to response-focused IER.

Numerous interpersonal benefits have been identified when IER occurs between people. Consistent with the affect theory of social exchange (Lawler, 2001), the emotional benefits of IER may serve to reinforce social bonds between the person attempting regulation and the target of their efforts. IER has been shown to increase trust both between individuals (Clark and Taraban, 1991; Niven et al., 2007, 2012) and between organizations (Williams, 2007) and can facilitate conflict resolution (Glinow et al., 2004). People who favor IER form more supportive social networks (Williams et al., 2018). Analyses of romantic couples has identified that IER improves relationship satisfaction (Gottman et al., 1998; Bloch et al., 2014; Levenson et al., 2014). The research in this area posits that interpersonal behaviors initiate a pattern of complementarity, such that positive interpersonal exchanges most often produce a positive behavioral response (Tracey, 1994; Losada and Heaphy, 2004), thus leading to improvements in the well-being of both the regulator and the target (Niven et al., 2009) and improving their relationship (Niven et al., 2012).

The researchers postulate the following hypotheses. Given that IER builds trust and improves relationships between people, we predict we will observe a similar effect of successful IER in consumption contexts in the form of improved customer-brand relationships. In particular, we hypothesize that antecedent-focused IER will result in increased brand trust and loyalty compared to response-focused IER, consistent with the process model of IER (Reeck et al., 2016). We anticipate specifically that antecedent-focused IER will result in more positive customer emotions and less negative customer emotions which is important as customer affect is a key precursor to customer loyalty (Jacoby and Chestnut, 1978; Oliver, 1999; Thomson et al., 2005; Evanschitzky et al., 2006; Iglesias et al., 2011), particularly after brand failures (Kalamas et al., 2008). These affective differences will subsequently enhance perceptions of the interaction with the employee implementing IER which in turn will improve brand loyalty and trust.

As an initial pretest for our hypothesis that IER improves customer perceptions of organizations, we analyzed a survey conducted in 2016 by the National Health Service of the United Kingdom (Picker Institute Europe, 2017) with over 46,000 respondents. The survey measured patients’ perceptions of the emotional support they received from hospital staff and their subsequent evaluations of their care and trust in the hospital. Patients’ evaluations of the emotional support they received from hospital staff and their evaluations of the service experience were highly correlated (*r* = 0.62, *p* < 0.001). Moreover, the level of emotional support provided was also correlated with the confidence and trust patients had in the hospital (*r* = 0.54, *p* < 0.001). These results provide initial support of the hypothesis that IER shapes perceptions of the service interaction and trust in the organization.

### Overview of Studies

The empirical work that follows examines several aspects of the influence of IER on brand outcomes. First, we show that antecedent-focused emotion regulation leads to better brand perceptions by changing the customers’ emotional response to the service interaction (Studies 1 and 2). Study 1 demonstrates that antecedent-focused emotion regulation improves customers’ subjective emotions and results in higher brand loyalty and brand trust ratings than response-focused IER. The influence of IER on brand outcomes is mediated by customer emotional responses and perceptions about the service interaction. Study 2 replicates this effect in a different service context and shows that, compared to a neutral control interaction, antecedent-focused strategies result in more positive and less negative feelings about the service interaction and increased perceptions of employee warmth. After establishing these key effects of IER strategies, in Study 3 we rule out an alternative explanation by explicitly noting the same benefits to customers in both the antecedent- and response-focused conditions. Study 3 shows that when the benefits of the service call are explicitly identical for both conditions, participants experienced less anger when the service representative employed antecedent-focused compared to response-focused regulation, leading to increased brand trust and loyalty. We also demonstrate that the positive effects of antecedent-focused IER extend to cases where a customer’s negative emotions arise for reasons unrelated to the brand (Study 4). We also establish a moderator of these effects, as customers who exhibit increased reactance to IER attempts are less likely to exhibit the benefits of IER. As we consistently demonstrate the negative effect of response-focused IER across Studies 1–4, in Study 5 we show that visibility can improve the outcome of a poor IER choice by increasing perceptions of benevolence. Our research contributes to the IER and customer relationship and brand management literatures by showing that different IER strategies influence consumer perceptions of the interaction with the employee and impact brand trust and loyalty through changing customer emotions. These findings extend understanding of IER in customer-employee relationships to encompass brand outcomes and suggest directly applicable strategies for consumer relationship and brand managers.

## Study 1

Study 1 offered an initial test of our hypothesis that antecedent-focused IER results in higher brand loyalty and brand trust ratings than response-focused IER. Specifically, we investigated the influence of the emotion regulation strategy employed by a customer service employee on brand loyalty and trust. As customers experience strong emotions in response to brand failures and their emotional reactions have consequences for their behavioral response to brand failures (Smith et al., 1999; Smith and Bolton, 2002; Bonifield and Cole, 2007; Kalamas et al., 2008), we first investigated the impact of IER in the context of a brand failure. We also examined whether participants’ emotional responses and evaluations of the service interaction mediated the effects of emotion regulation strategy on brand loyalty and trust.

### Methods

#### Participants and Procedure

308 business students at a northeastern university participated in an online study in exchange for course credit. Participants were required to be between the ages of 18 and 65. 53 participants were removed from analyses for failing attention checks, leaving a final sample of 255 (99 female, mean age 19.9 years old, see Supplementary Table 1 for additional demographics). After providing informed consent, participants were told to imagine themselves in a situation where their new laptop gave an error message and turned off while they were working on a project due soon and they called customer support. Participants read the service representative’s initial response, which was manipulated across two between-subjects conditions (see Supplementary Material). In the antecedent-focused condition, the representative attempts to reframe the situation by telling the customer a beneficial aspect of the situation (“It’s good that you called in, since we can also make sure your virus protection software is running properly”), whereas in the response-focused condition, the representative attempts to alter the outward display of the customer’s reaction (”Please take a deep breath and calm down”). All participants were randomly assigned to conditions. All measures, manipulations, and exclusions are disclosed. A sensitivity analysis revealed this experiment was sufficiently powered to detect an effect size of *d* = 0.35 or higher. Data were analyzed using *t*-tests and analyses of mediation models.

#### Measures

After reading the service representative’s response, participants then rated the customer service interaction, their feelings after the interaction, their perceptions of the service representative, and their brand loyalty and trust. Ratings about the interaction were measured with the item “How would you rate your interaction with the customer service representative?” (from 1 = “extremely negative” to 7 = “extremely positive”). Feelings about the service interaction were measured by a short version of the PANAS scale (Watson et al., 1988) with seven items (Happy, α = 0.90; Angry, α = 0.89; from 1 = “not at all” to 7 = “extremely”). To measure brand loyalty, we used five items from the service loyalty scale (Gremler and Brown, 1996) including items like “I would not consider switching away from this brand” (α = 0.89; 1 = “strongly disagree” to 7 = “strongly agree”). For brand trust, a 7-item (α = 0.88) scale (Munuera-Aleman et al., 2003) was adapted to match the study design with items like “I could rely on this brand to solve the problem” (from 1 = “strongly disagree” to 7 = “strongly agree”).

### Results and Discussion

#### Feelings About Service Interaction

**FIGURE 1:**
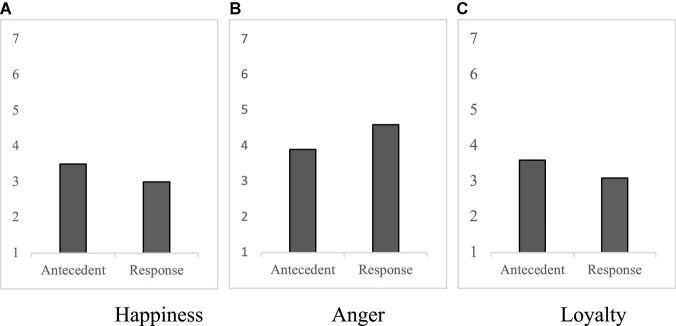
Means for happiness **(A)**, anger **(B)**, and brand loyalty **(C)** in Study 1.

An independent samples *t*-test was conducted to compare the effect of the service representative’s IER approach on participants’ feelings after the interaction. There was a significant effect of emotion regulation on feelings of happiness about the service interaction, with greater reported happiness in the antecedent-focused condition compared to the response-focused condition [*M*_ant_ = 3.5, *M*_resp_ = 3.0, *t*(253) = 2.72, *p* = 0.007, *d* = 0.34, Figure 1A]. Similarly, the antecedent-focused condition resulted in significantly lower anger than the response-focused condition [*M*_ant_ = 3.9, *M*_resp_ = 4.6, *t*(253) = 3.90, *p* < 0.001, *d* = 0.48, Figure 1B]. Given that anger in response to brand failures predicts key consumer behaviors such as negative word-of-mouth and repurchase intention (Bonifield and Cole, 2007; Kalamas et al., 2008), this observed reduction in anger is important. These findings are consistent with the notion that antecedent-focused IER, by focusing on earlier stages of the emotion generation process, is more effective at altering emotional responses than response-focused IER (Reeck et al., 2016).

#### Brand and Employee Related Outcomes

We next examined how the customer service representatives IER strategy altered perceptions of the brand and the employee. There was a significant effect of emotion regulation on brand loyalty, with higher loyalty in the antecedent-focused condition compared to the response-focused condition [*M*_ant_ = 3.6, *M*_resp_ = 3.1, *t*(253) = 3.00, *p* = 0.003, *d* = 0.38, Figure 1C]. Similarly, there were significant differences in brand trust across conditions, as the antecedent-focused condition resulted in higher trust than the response-focused condition [*M*_ant_ = 3.1, *M*_resp_ = 2.6, *t*(253) = 4.48, *p* < 0.001, *d* = 0.56]. Antecedent focused strategies produced a positive impact on ratings about the service interaction as well. Participants rated the interaction more positively [*M*_ant_ = 4.8, *M*_resp_ = 4.2, *t*(253) = 3.78, *p* < 0.001, *d* = 0.47] in the antecedent-focused condition.

(A) Happiness, (B) Anger, (C) Loyalty.

#### Process Behind Influence of Interpersonal Emotion Regulation on Brand Outcomes

**FIGURE 2:**
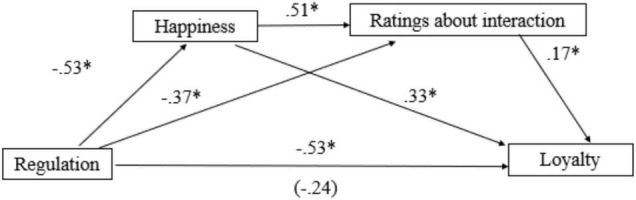
Interpersonal emotion regulation increases loyalty through changing emotional responses toward the service interaction in Study 1. *p < 0.05 (Value in parentheses indicates the effect of emotion regulation on the dependent variable after controlling for the mediators).

We predicted that IER enhances brand loyalty by improving the customer’s emotional state, which in turn boosts positive evaluations of the service interaction. Consistent with this prediction, a serial mediation model (Hayes, 2013) revealed that antecedent-focused emotion regulation (variable coded as antecedent-focused = 1, response-focused = 2) resulted in higher brand loyalty because this emotion regulation approach increased participants’ happiness [*B* = −0.53, SE = 0.19, *t*(253) = 2.71, *p* = 0.007], which in turn resulted in positive evaluations of the service interaction [*B* = 0.51, SE = 0.05, *t*(252) = 11.73, *p* < 0.001] and subsequently increased loyalty toward the brand (95% CI for the indirect effect = −0.48, −0.11; Figure 2).

A similar serial mediation model revealed that antecedent-focused emotion regulation also resulted in higher brand trust (95% CI for the indirect effect, −0.32 to −0.08; see Supplementary Figure 1) because the emotion regulation strategy increased happiness about the interaction with the service employee [*B* = −0.53, SE = 0.19, *t*(253) = 2.71, *p* = 0.007] which in turn resulted in positive evaluations about the service interaction [*B* = 0.51, SE = 0.05, *t*(252) = 11.73, *p* < 0.001]. In both serial mediations, the indirect effect becomes insignificant when the location of intermediate measures is reversed, showing that increased happiness leads to positive evaluations of service interaction, not the opposite.

We observed the same serial mediation relationship when anger is the first mediator and ratings about the interaction as the second one. Antecedent focused strategies resulted in increased brand loyalty and trust through reducing anger which in turn resulted in better evaluations of the service interaction (95% CI for the indirect effect = −0.62 to −0.21 for brand loyalty; −0.4 to −0.15 for brand trust). These results indicate that antecedent-focused IER changes the emotional response toward the service interaction resulting in better evaluations of the interaction which in turn positively influences brand outcomes.

Study 1 provided initial evidence of the effect of emotion regulation on customers’ emotions and the mechanism through which IER influences brand trust and loyalty. We found that antecedent-focused emotion regulation presents a better way to deal with customers feeling negative emotions in service interactions by changing the emotional response to the service interaction. However, several important limitations of Study 1 should be noted. First, the scenario used in the study concerned a specific service problem which involved product failure. Thus, it is necessary to replicate the findings in a service context to test the generalizability of the results we obtained. Second, the absence of a control condition limits the inferences we can make with regards to the effect of different emotion regulation strategies. For example, it is unclear if the differences observed in Study 1 are due to improvements following antecedent-focused emotion regulation, detriments following response-focused emotion regulation, or both. We address both of these limitations in Study 2.

## Study 2

Our central hypothesis is that IER results in increased brand loyalty and trust through feelings about the service interaction and positive evaluations of the service employee. In Study 2 we aimed to conceptually replicate the results from Study 1 in a different service context in which instead of an actual problem with the product, the consumer is upset about the service provided by the company. Additionally, we included a control condition to examine how both IER conditions differ from a neutral response. Lastly, we measured perceptions of employee warmth and competence (Fiske et al., 2007) to augment analyses of the impact of IER on employee evaluations. It might be the case that antecedent-focused IER improves brand loyalty and trust through a halo effect, such that all aspects of the interaction are viewed more positively. However, we believe this explanation is unlikely. Instead, we expect that antecedent-focused IER is more likely to influence judgments related to the benevolence of the employee and the brand, such as warmth, and unlikely to influence unrelated judgments, such as perceptions of competence.

### Methods

#### Participants and Procedure

538 business students at a northeastern university participated in an online study in exchange for course credit. Participants were required to be between the ages of 18 and 65. 195 participants were removed for failing attention checks and 35 people were removed for completing the survey either too fast (less than 3 min) or too slow (more than 2,000 s). The final sample was 308 participants (141 female, mean age 20.5 years old, see Supplementary Table 1 for additional demographics). After providing informed consent, participants were told to imagine themselves in a situation where an unjustified overdraft fee was charged on their bank account and they called the bank’s customer service to address the issue. Participants read the service representative’s initial response, which was manipulated across three between-subjects conditions (see Supplementary Material). In the antecedent-focused condition, the representative attempts to reframe the situation (“It’s good that you called in, since we can also make sure all the other information is correct on your account today”), whereas in the response-focused condition, the representative attempts to alter the outward display of the customer’s reaction (“Please take a deep breath and calm down”). A third condition served as a control in which emotion regulation was not attempted (“I’m sorry for the problem, and I will be able to fix it”). Participants were randomly assigned to conditions. The same measures used in Study 1 were employed in Study 2. Additionally, for perceptions of the service employee, a 4-item warmth-competence scale (Fiske et al., 2007) was used (Warmth, α = 0.92, Competence α = 0.93). A sensitivity analysis revealed this experiment was sufficiently powered to detect an effect size of *d* = 0.17 or higher. Data were analyzed using *t*-tests, ANOVAs, and analyses of mediation models.

### Results and Discussion

#### Feelings About Service Interaction

A one-way between-subjects ANOVA was conducted to compare the effect of the service representative’s IER approach on participants’ feelings after the interaction. There was a significant effect of emotion regulation on feelings of happiness about the customer service interaction [*F*(2, 305) = 5.57, *p* = 0.004, η_p_^2^ = 0.04]. *Post hoc* comparisons indicated greater reported happiness in the antecedent-focused condition compared to both the response-focused [*M*_ant_ = 5.4, *M*_resp_ = 4.8, *t*(199) = 2.73, *p* = 0.007, *d* = 0.38] and control conditions [*M*_con_ = 4.7, *t*(199) = 3.12, *p* = 0.002, *d* = 0.43]. Similarly, a one-way ANOVA for feelings of anger revealed significant differences across conditions [*F*(2, 305) = 6.46, *p* = 0.002, η_p_^2^ = 0.04], as the antecedent-focused condition resulted in lower anger than the response-focused [*M*_ant_ = 2.3, *M*_resp_ = 3.1, *t*(199) = 3.64, *p* < 0.001, *d* = 0.51] and control conditions [*M*_con_ = 2.9, *t*(199) = 2.40, *p* = 0.017, *d* = 0.33]. Importantly, happiness [*M*_cont_ = 4.7, *M*_resp_ = 4.8, *t*(212) = 0.49, *p* = 0.624, *d* = 0.06] and anger [*M*_cont_ = 2.9, *M*_resp_ = 3.1, *t*(212) = 1.14, *p* = 0.255, *d* = 0.15] did not differ between the response-focused and the control conditions. Thus, antecedent-focused IER results in less negative and more positive emotional responses compared to both a neutral control condition and response-focused IER. However, employing a response-focused IER strategy does not appear to be worse than not attempting any IER.

#### Brand and Employee Related Outcomes

We next examined how the customer service representatives’ IER strategy altered perceptions of the brand and the employee. There was a significant effect of emotion regulation condition on brand loyalty [*F*(2, 305) = 3.07, *p* = 0.048, η_p_^2^ = 0.02]. *Post hoc* comparisons indicated higher loyalty in the antecedent-focused condition compared to the control condition [*M*_ant_ = 5.1, *M*_con_ = 4.7, *t*(199) = 2.42, *p* = 0.016, *d* = 0.34], but the comparison of the antecedent- and response-focused conditions was not significant [*M*_res_ = 4.9, *t*(199) = 1.11, *p* = 0.268, *d* = 0.15]. Brand loyalty did not significantly differ between the control and response-focused conditions [*t*(212) = 1.40, *p* = 0.164]. For brand trust, there was not a significant effect of emotion regulation condition in the main ANOVA [*F*(2, 305) = 1.76, *p* = 0.175]. However, an independent samples *t*-test revealed trend level differences in trust ratings across conditions, showing that the antecedent-focused condition resulted in higher trust ratings compared to the control condition [*M*_ant_ = 3.7, *M*_cont_ = 3.5, *t*(199) = 1.80, *p* = 0.073, *d* = 0.25]. There was not a significant difference in brand trust between the response-focused and control conditions [*M*_res_ = 3.7, *t*(212) = 1.16, *p* = 0.247] or between the response-focused and antecedent-focused conditions [*t*(199) = 0.72, *p* = 0.472].

We next examined perceptions of the customer service agent. One possibility is that antecedent-focused regulation produces a halo effect, in which all aspects of the interaction (including the employee) are rated more positively. However, we do not predict this pattern. While we expect to observe that antecedent-focused IER enhances perceptions of the customer service employee’s warmth, we predict that there will be no differences between conditions in competence, since benevolent intentions on the part of the customer service employee would not impact their perceived abilities. Confirming our predictions, a one-way ANOVA for perceptions of the service employee revealed significant differences across conditions [*F*(2, 307) = 4.39, *p* = 0.013, η_p_^2^ = 0.03], as the antecedent-focused condition resulted in higher perceptions of warmth than the response-focused [*M*_ant_ = 5.5, *M*_resp_ = 5.1, *t*(199) = 2.14, *p* = 0.033, *d* = 0.29] and the control conditions [*M*_ant_ = 5.5, *M*_cont_ = 4.9, *t*(199) = 3.05, *p* = 0.003, *d* = 0.42]. The control and response-focused conditions did not differ from each other [*t*(212) = 0.75, *p* = 0.45, *d* = 0.10]. Importantly, no differences in competence were observed across conditions [*F*(2, 307) = 1.33, *p* = 0.265]. Thus, antecedent-focused IER does not result in a halo effect of judgments but rather is specific to judgments of warmth, likely due to the benevolence signaled by attempting IER using an antecedent-focused regulatory strategy.

#### Process Behind Influence of Interpersonal Emotion Regulation on Brand Outcomes

We observed the same serial mediation relationship as in Study 1, revealing that the antecedent-focused emotion regulation resulted in higher brand loyalty because this emotion regulation approach increased participants’ happiness [*B* = −0.63, SE = 0.23, *t*(199) = 2.71, *p* = 0.007], which in turn resulted in positive evaluations of the service interaction [*B* = 0.65, SE = 0.06, *t*(198) = 11.72, *p* < 0.001] and subsequently increased loyalty toward the brand as a whole (95% CI for the indirect effect = −0.41, −0.05; Supplementary Figure 2).

A similar serial mediation model revealed that antecedent-focused emotion regulation also resulted in higher brand trust (95% CI for the indirect effect = −0.3, −0.03; Supplementary Figure 3) because the emotion regulation strategy increased happiness about the interaction with the service employee [*B* = −0.63, SE = 0.23, *t*(199) = 2.72, *p* = 0.007] which in turn resulted in positive evaluations about the service interaction [*B* = 0.65, SE = 0.06, *t*(198) = 11.72, *p* < 0.001]. Replacing happiness with anger also produced qualitatively similar results pointing to the emotional response as the underlying mechanism.

Study 2 replicated the findings of Study 1 in a different service setting and showed that, compared to a neutral control interaction, antecedent-focused strategies result in more positive and less negative feelings about the service interaction and increased perceptions of employee warmth. This study also demonstrates that antecedent-focused strategies improve reactions to the interaction and brand outcomes compared not only to response-focused emotion regulation but also to a lack of attempted IER. Interestingly, the response-focused condition did not differ in reactions to the interaction or brand outcomes from the control condition in which IER was not attempted. Like Study 1, these results indicate that antecedent-focused IER changes the emotional response toward the service interaction resulting in better employee evaluations which in turn positively influences brand outcomes.

## Study 3

The results of the first two experiments demonstrate that antecedent-focused IER changes the customer emotional response toward the service interaction and leads to better employee and service ratings resulting in more positive brand evaluations. In Study 3, we expand these findings by ruling out a possible alternative explanation for the findings we presented so far. Antecedent-focused IER in Studies 1–2 involved reframing the situation to create a cognitive change and thereby alter emotional responses. Specifically, by offering benefits such as checking whether the virus protection software is up-to-date (Study 1) or updating the information on the account (Study 2), the service representative attempts to have the customer reappraise the situation. However, it is possible that the reappraisal attempt itself may create a perception of an additional benefit which is lacking in the response-focused condition. It is possible that not the choice of emotion regulation strategy, but this additional benefit may drive the positive employee and brand perceptions we observe in antecedent-focused conditions in these studies. In Study 3, we address this issue by explicitly noting the same benefits in both the antecedent- and response-focused conditions. We expect to observe the same pattern of results as in Studies 1–2.

### Methods

#### Participants and Procedure

351 adults (138 female, mean age 34.8 years old, see Supplementary Table 1 for additional demographics) recruited through Amazon Mechanical Turk participated in an online study in exchange for $1. Participants were required to be between the ages of 18 and 65 and to have completed at least 50 prior HITs. The same manipulation in Study 1 was used with a slight modification to both conditions by including a sentence (“While solving the main problem, the representative also confirms your virus protection software is up to date”) to make sure all participants have the same benefit in addition to having their primary problem solved. This sentence is placed after the emotion regulation manipulations. All participants were randomly assigned to the two conditions (antecedent-focused vs. response-focused). After reading the salesperson’s response, participants responded to the questions measuring the same dependent variables as in the previous experiments. A sensitivity analysis revealed this experiment was sufficiently powered to detect an effect size of *d* = 0.29 or higher. Data were analyzed using *t*-tests and analyses of mediation models.

### Results and Discussion

#### Feelings About Service Interaction

An independent samples *t*-test was conducted to compare the effect of emotion regulation on how participants feel after the service interaction. The effect of emotion regulation on feelings of happiness about the service interaction was not significantly different across conditions [*M*_ant_ = 4.9, *M*_resp_ = 4.7, *t*(349) = 1.43, *p* = 0.162]. When the benefits of the call are explicit in both conditions, participants experience similar levels of happiness. However, a similar *t*-test for feelings of anger showed significant differences across conditions, as the antecedent-focused condition resulted in lower self-reported anger than the response-focused condition [*M*_ant_ = 2.5, *M*_resp_ = 2.9 *t*(349) = 2.14, *p* = 0.033, *d* = 0.22]. Given that negative emotions are central to many customer service interactions (Menon and Dubé, 2007; Little et al., 2013) involving product failure like the scenario included here and that anger following brand failures predicts subsequent retaliatory behaviors by consumers (Bonifield and Cole, 2007; Kalamas et al., 2008), demonstrating this reduction in negative emotion following antecedent-focused IER is highly relevant.

#### Brand and Employee Related Outcomes

Antecedent-focused emotion regulation resulted in higher brand loyalty compared to the response-focused condition [*M*_ant_ = 4.4, *M*_resp_ = 4.1, *t*(349) = 2.16, *p* = 0.031, *d* = 0.23]. Similarly, antecedent-focused IER resulted in marginally higher brand trust than the response-focused condition [*M*_ant_ = 3.3, *M*_resp_ = 3.2, *t*(349) = 1.68, *p* = 0.093].

Antecedent-focused strategies revealed a positive impact on perceptions of the employee as well. Participants rated the customer service representative as warmer in the antecedent-focused condition compared to the response-focused condition [*M*_ant_ = 5.3, *M*_resp_ = 5.0, *t*(349) = 2.06, *p* = 0.040, *d* = 0.21]. Competence ratings were not significantly different across conditions [*t*(349) = 0.41, *p* = 0.662], consistent with the findings from Study 2.

#### Process Behind Influence of Interpersonal Emotion Regulation on Brand Outcomes

When the material benefits of the customer service call were explicitly identical between the two conditions, antecedent-focused IER did not produce significantly higher happiness ratings, perhaps due to the generally high levels of happiness experienced by participants in both conditions. Importantly, however, antecedent-focused regulation did reduce feelings of anger about the service interaction. Consistent with the results we obtained in Studies 1–2, a serial mediation model revealed that the antecedent-focused emotion regulation resulted in higher brand loyalty because antecedent-focused emotion regulation decreased participants’ feelings of anger [*B* = 0.38, SE = 0.18, *t*(349) = 2.14, *p* = 0.033], which in turn resulted in positive evaluations of the interaction [*B* = −0.34, SE = 0.04, *t*(349) = 8.21, *p* < 0.001] and subsequently increased loyalty toward the brand as a whole (95% CI for the indirect effect = −0.36, −0.005; see Supplementary Figure 4). The same serial mediation relationship emerges when trust is the dependent variable in the model (95% CI for the indirect effect = −0.21, −0.002).

Taken as a whole, Study 3 demonstrated that IER strategies influence brand loyalty and trust by altering customer emotions and subsequent judgments of the service interaction. When the benefits of the service call are explicitly identical for both conditions, participants still experienced less anger when the service representative employed antecedent-focused compared to response-focused regulation. As mitigating negative emotions is often the principal goal of emotion regulation attempts by customer service representatives (Little et al., 2013), this finding is of key importance. Importantly, this reduced anger leads to improved perceptions of the service interaction and, subsequently, increased brand loyalty and brand trust.

## Study 4

Studies 1–3 consistently demonstrate that antecedent-focused IER results in improved outcomes for brands compared to response-focused IER. These improvements arise as consumers feel less negative emotion and more positive emotion following the IER, which in turn improves their perceptions of the interaction and their attitudes toward the brand. In Studies 1–3, the negative emotion that the customer service representative was attempting to regulate arose for reasons related to the brand, such as dissatisfaction with the product or service. In Study 4, we wanted to examine whether differences in IER would influence brand outcomes even when negative emotions arose for reasons unrelated to the brand. Consumers might experience negative emotions for a range of personal reasons, including making difficult decisions (Luce, 1998; Luce et al., 2001; Yi and Baumgartner, 2004), and brand representatives such as salespeople may attempt to help them manage those negative feelings. As antecedent-focused IER improves consumers’ emotional state and subsequent perceptions of the interaction with implications for brand outcomes, brand benefits may still emerge even when the feelings being targeted by IER arise for reasons unrelated to the brand.

In the case of emotions arising for personal reasons, however, it is possible that some consumers may perceive the IER attempt as an intrusion and thus develop reactance toward it. Reactance is a motivational state aimed at restoring the loss of freedom resulting from a perception of a threat to one’s autonomy (Hong and Faedda, 1996). When an employee attempts to manage customer feelings that arose for reasons unrelated to the brand, there is the risk that this attempt will be viewed as an intrusion. If this is the case, the attempted IER may engender reactance and the brand would not experience the benefits that would typically emerge from antecedent-focused IER. In such circumstances, reactance would be a key moderator of the effect of regulatory strategy on brand outcomes.

To test these predictions, in Study 4 we examined whether the positive effects of antecedent-focused IER extend to cases where a customer’s negative emotions arise for reasons unrelated to the brand. We anticipate that reactance will serve as a moderator of the effect of IER strategy on brand outcomes.

### Methods

#### Participants and Procedure

Participants in the experiment were 202 adults recruited through Amazon Mechanical Turk to participate in the study in exchange for $1. Participants were required to be between the ages of 18 and 65 and to have completed at least 50 prior HITs. 4 participants failed attention checks and were removed from analysis, leaving a final sample of 198 (75 female, mean age 34.1 years old). After providing informed consent, all participants were told to imagine a scenario where they feel upset because of a personal issue while they were shopping for a shirt. Participants read the service representative’s initial response, which was manipulated across two between-subjects conditions: the service representative employed either antecedent-focused (“In these situations, it sometimes helps me to remember it’s just one bad day and overall my friends care about me”) or response-focused IER (“In these situations, it sometimes helps me to calm myself down by taking long, deep breaths and trying to smile more,” see Supplementary Material). All participants were randomly assigned to conditions.

After reading the salesperson’s response, in addition to the dependent variables in the previous experiments, we measured reactance toward the emotion regulation attempt. We used a modified version of the Hong and Faedda (1996) reactance scale, including items like “I considered the salesperson’s advice as an intrusion” (α = 0.95; from 1 = “strongly disagree” to 7 = “strongly agree”). A sensitivity analysis revealed this experiment was sufficiently powered to detect an effect size of *d* = 0.4 or higher. Data were analyzed using *t*-tests and analyses of mediation models.

### Results and Discussion

#### Feelings About Service Interaction

Consistent with the findings from Studies 1–2, there was a significant effect of emotion regulation on feelings of happiness about the service interaction with greater reported happiness in the antecedent-focused condition compared to the response-focused condition [*M*_ant_ = 4.5, *M*_resp_ = 3.9, *t*(196) = 3.05, *p* = 0.003, *d* = 0.63]. We didn’t find a significant effect of emotion regulation on feelings of anger [*M*_ant_ = 1.7, *M*_resp_ = 1.9, *t*(196) = 0.65, *p* = 0.513, *d* = 0.09]. The absence of an effect of IER on anger may be due to the fact that anger was less likely to be experienced in this scenario, given that it did not feature a product or service failure.

#### Brand and Employee Related Outcomes

As observed in our previous studies, antecedent-focused regulation produced significantly higher ratings of brand loyalty and positive perceptions of the service employee. Antecedent-focused regulation resulted in higher loyalty ratings than response-focused regulation [*M*_ant_ = 5.7, *M*_resp_ = 5.2, *t*(196) = 2.69, *p* = 0.008, *d* = 0.81]. Similarly, there was a significant effect of antecedent-focused emotion regulation on brand trust as the antecedent-focused condition resulted in higher trust than the response-focused condition [*M*_ant_ = 4.2, *M*_resp_ = 3.9, *t*(196) = 2.18, *p* = 0.030, *d* = 0.59]. Thus, IER can improve customer’s relationship with the brand even when emotions arise for reasons unrelated to the brand.

In terms of employee perceptions, participants rated the customer service representative marginally warmer in the antecedent-focused condition compared to the response-focused condition [*M*_ant_ = 6.0, *M*_resp_ = 5.7, *t*(196) = 1.75, *p* = 0.081, *d* = 0.85]. As in previous experiments, antecedent-focused emotion regulation had a significant effect on evaluations of the service interaction with more positive evaluations in the antecedent-focused condition compared to the response-focused condition [*M*_ant_ = 6.1, *M*_resp_ = 5.5, *t*(196) = 3.28, *p* = 0.001, *d* = 0.25]. We observed the same process behind the influence of IER on brand outcomes. Specifically, antecedent-focused emotion regulation results in increased brand loyalty by strengthening happiness about the service interaction, which in turn boosts positive evaluation of the service interaction (95% CI for the indirect effect, −0.7 to −0.14). We observed the same serial mediation when trust is the dependent variable (95% CI for the indirect effect, −0.33 to −0.9).

#### Reactance Moderates the Influence of Interpersonal Emotion Regulation on Brand Outcomes

**FIGURE 3:**
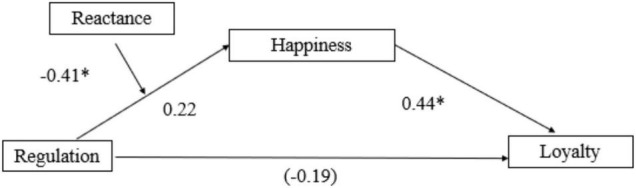
Interpersonal emotion regulation increases loyalty through changing emotional responses toward the service interaction in Study 4. This relationship is moderated by reactance toward regulation *p < 0.05 (Value in parentheses indicates the effect of emotion regulation on the dependent variable after controlling for the mediators).

We predicted that the effect of emotion regulation on brand loyalty is mediated by happiness, which in turn is moderated by the reactance toward the emotion regulation strategy. The moderated mediation model (Hayes, 2013) showed that the direct effect of emotion regulation on brand loyalty is not significant [*B* = −0.19, SE = 0.14, *t*(194) = 1.24, *p* = 0.216], but the indirect effect through happiness about the service interaction was significant (index of moderated mediation = −0.18, with a 95% CI for the indirect effect, −0.34 to −0.03, Figure 3). Conditional indirect effects of emotion regulation on loyalty at different levels of reactance showed that, when reactance toward emotion regulation strategy is low the indirect effect is stronger on loyalty. That is, people who experienced less reactance to the service representative’s attempt to manage their emotions experienced stronger effects of IER on their emotional state and, subsequently, their brand loyalty. We found a similar pattern in trust such that the direct effect of emotion regulation on brand trust is not significant [*B* = −0.09, SE = 0.09, *t*(194) = 1.08, *p* = 0.281], but the indirect effect through happiness about the service interaction was significant (index of moderated mediation = −0.07, with a 95% CI for the indirect effect, −0.13 to −0.01). Similarly, reactance moderated the influence of emotion regulation on trust.

Study 4 showed that IER improves brand evaluations even when the customer’s negative emotions arise for reasons unrelated to the products or services that the company offers. This finding indicates that the benefits of IER can extend to a broader range of circumstances in which customers experience unwanted feelings beyond merely product or service failure. The key mechanism of the effect appears to be the customer service representative’s influence on the customer’s feelings following the interaction. Importantly, this finding is moderated by reactance, such that the influence of IER on happiness and brand loyalty is strongest for people who experience less reactance to the service representative’s attempt to manage their feelings.

## Study 5

In Studies 1–4, we demonstrate that response-focused IER results in more negative customer feelings, less positive evaluations of the interaction, and lower brand loyalty and trust than antecedent-focused IER. Given that employees tend to select this poor regulation strategy when consumers express high levels of negative emotion (Little et al., 2013), we next sought to find a way to improve outcomes associated with response-focused IER. Toward this end, we manipulated visibility (Bolger and Amarel, 2007)—customers’ awareness of the IER attempt. As suggested by the process model of IER (Reeck et al., 2016), we hypothesized that visibility improves brand outcomes by increasing the customer’s perceptions of benevolence on the part of the service representative and, by extension, the brand. Perceptions of benevolence are relevant for the classification of relationships as communal or exchange (Clark and Mills, 1979, 1993; Clark et al., 1987). In communal relationships, benefits are given in response to needs to demonstrate concern for the other person, whereas in exchange relationships benefits are given with the expectation of receiving a comparable benefit. We anticipate that when consumers are aware of the IER attempt, they are more likely to see their relationship with the brand as communal and attribute benevolent intentions to the brand and service representative. We test this proposition in Study 5, examining whether making the attempt to manage the customer’s emotions visible will improve outcomes associated with response-focused IER strategies.

### Methods

#### Participants and Procedure

Totally 559 adults were recruited through Amazon Mechanical Turk participated in an online study in exchange for $1. Participants were required to be between the ages of 18 and 65 and to have completed at least 50 prior HITs. 61 participants failed attention checks and their data were removed, leaving a final sample of 498 adults (254 female, mean age 35.2 years old, see Supplementary Table 1 for additional demographics). We designed a 2 (antecedent-focused vs. response-focused) × 2 (visible vs. invisible) between-subjects experiment with a control condition. The same manipulation in Study 2 was used with a slight modification in visible conditions with the inclusion of a sentence making the IER attempt explicit for the participant (“I can tell you are upset, and part of my job is to help you feel better”). All participants were randomly assigned to conditions.

In addition to the dependent variables measured in Studies 1–3, we measured benevolence using perceptions of the service interaction. Participants evaluated the benevolence of the service employee with responses to the question “The customer service representative tried to make you feel better” (from 1 = “strongly disagree” to 7 = “strongly agree”). A sensitivity analysis revealed this experiment was sufficiently powered to detect an effect size of *d* = 0.15 or higher. Data were analyzed using *t*-tests, ANOVAs, and analyses of mediation models.

### Results and Discussion

#### Visibility

**FIGURE 4:**
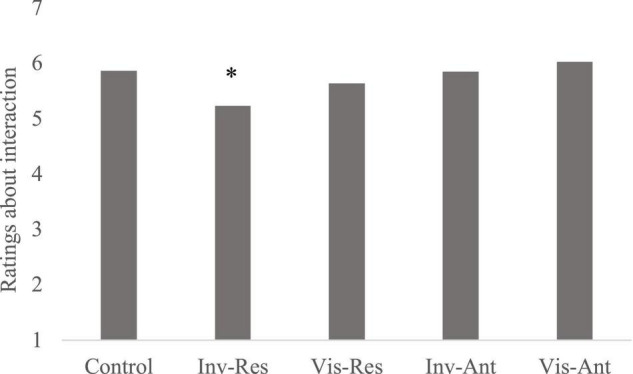
Means for ratings about service interaction ratings in Study 5. *p < 0.05.

A one-way ANOVA for ratings about the service interaction showed significant differences across conditions [*F*(4, 493) = 5.01, *p* = 0.001, η_p_^2^ = 0.04, Figure 4]. *Post hoc* comparisons showed that invisible response-focused emotion regulation (M_invis–res_ = 5.2) resulted in significantly lower ratings about the interaction compared to all other conditions visible response-focused [M_vis–res_ = 5.6, *t*(197) = 1.91, *p* = 0.057, *d* = 0.26], control [M_cont_ = 5.8, *t*(197) = 3.07, *p* = 0.002, *d* = 0.43], invisible antecedent-focused [M_invis–ant_ = 5.8, *t*(197) = 2.98, *p* = 0.003, *d* = 0.42], and visible antecedent-focused conditions [M_vis–ant_ = 6.0, *t*(197) = 3.75, *p* < 0.001, *d* = 0.53]. When we pool all responses together and remove control cases we see a main effect of visibility on brand loyalty [M_vis_ = 5.3, M_invis_ = 5.0, *F*(1, 396) = 4.71, *p* = 0.030, η_p_^2^ = 0.01], with better evaluations in visible compared to invisible emotion regulation conditions. We observed the same result in brand trust [M_vis_ = 3.7, M_invis_ = 3.5, *F*(1, 396) = 4.19, *p* = 0.041 η_p_^2^ = 0.01]. When we include the control cases we see a main effect of visibility on perceptions of the relationship [*F*(1, 495) = 8.33, *p* = 0.004, η_p_^2^ = 0.03]. *Post hoc* comparisons indicated greater perception of benevolence in the visible condition compared to both the invisible [M_vis_ = 6.0, M_invis_ = 5.5, *t*(393.3) = 3.54, *p* < 0.001, *d* = 0.35] and control conditions [M_cont_ = 5.4, *t*(298) = 3.34, *p* = 0.001, *d* = 0.40]. These findings show that visibility improves a poor strategy choice by the service representative.

#### Process Behind Influence of Interpersonal Emotion Regulation on Brand Outcomes

We replicated the findings of the previous experiments by conducting the same serial mediation analysis in the invisible conditions with feelings of happiness and ratings about interaction as the serial mediators (95% CI for the indirect effect—brand trust: −0.41 to −0.04; brand loyalty: −0.62 to −0.07).

**FIGURE 5:**
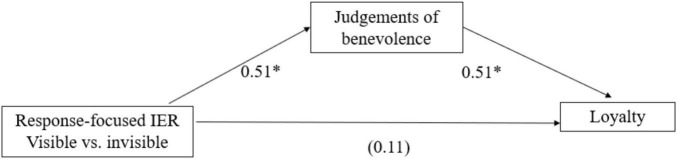
The mediating role of judgments of benevolence in Study 5. *p < 0.05.

To see the underlying mechanism behind the visibility of IER we ran an additional mediation analysis with only response-focused cases. We conducted the analysis with bootstrapping (Hayes, 2013) focusing on the response-focused conditions to examine the influence of visibility on judgments of benevolence and brand loyalty. The results revealed that the direct effect of visibility on brand loyalty is not significant [*B* = 0.11, SE = 0.15, *t*(197) = 0.73, *p* = 0.464], but the indirect effect through judgments of benevolence was significant (Indirect effect = 0.26, with a 95% CI for the indirect effect, 0.04–0.49, Figure 5). Thus, visibility of response-focused IER influences brand loyalty through judgments of benevolence. We observe the same mediation when we replace the brand loyalty with trust as the dependent variable (Indirect effect = 0.17, with a 95% CI for the indirect effect, 0.03–0.32).

In Study 5 we showed that visibility can improve the outcome of a poor IER choice by increasing perceptions of benevolence. Making a response-focused regulation attempt visible resulted in higher perceptions of benevolence on the part of the service representative and the brand, which in turn lead to higher judgments of brand loyalty and brand trust. Enhancing the visibility of regulation attempts can thus buffer brands from the negative consequences typically associated with response-focused IER attempts.

## General Discussion

The IER strategy frontline employees utilize to manage customers’ emotions not only influences how consumers feel but also how they evaluate the interaction and, ultimately, the brand. Five studies support our prediction that antecedent-focused IER results in more positive emotions, better interaction evaluations, and higher brand loyalty and trust ratings compared to response-focused IER. The IER strategy implemented by the employee changes the consumer emotional response to the service interaction resulting in better service evaluations which in turn positively influence brand loyalty and trust. Importantly, these findings were observed not only when consumer emotions arose due to a brand failure, but also when consumers were experiencing emotions for reasons unrelated to the brand. When the customer’s negative emotion originates for reasons unrelated to the brand, reactance moderates the influence of IER on brand outcomes. For customers with low (high) reactance to the interpersonal regulation attempt, brand trust and loyalty evaluations are more (less) favorable. Additionally, we demonstrate that enhancing the visibility of IER attempts serves as an important boundary condition. When a response-focused IER attempt is made explicit to the consumer, the consumer subsequently evaluates the brand similarly to when an antecedent-focused IER strategy is implemented. The benefits of making IER attempts visible are mediated by increased perceptions of the benevolence of the employee. Taken as a whole, these findings demonstrate that antecedent-focused IER can result in positive emotional benefits for the consumer and subsequent enhancements in brand trust and brand loyalty.

The present article is the first demonstration of the positive relationship between antecedent-focused IER and brand outcomes. We illustrate a causal pathway that ties together employee IER strategies, customer emotional responses, service interaction evaluations, and brand trust and loyalty. These findings build on previous theories of emotion regulation. Previous research has demonstrated that antecedent-focused emotion regulation results in more adaptive outcomes than response-focused emotion regulation when people attempt to manage their own feelings, including improved subjective experience (Gross and Levenson, 1993; Gross, 1998a; Ray et al., 2010; Brans et al., 2013), reduced physiological responding (Gross and Levenson, 1993; Gross, 1998a; Richards and Gross, 2000; Ochsner et al., 2004; Goldin et al., 2008; Ochsner and Gross, 2008), enhanced memory for emotional events (Richards and Gross, 2000; Richards et al., 2003; Hayes et al., 2010), and better social relationships (Gross and John, 2003; Srivastava et al., 2009; English et al., 2012; Finkel et al., 2013). Here, we demonstrate related benefits when antecedent-focused compared to response-focused emotion regulation strategies are deployed to attempt to manage another person’s feelings rather than one’s own. These findings are consistent with predictions from the process model of IER (Reeck et al., 2016), which posits that antecedent-focused IER should result in improved outcomes compared to response-focused IER. The current findings also complement prior research focused on IER, which demonstrate interpersonal benefits following successful IER between people, such as improved relationship satisfaction and increased trust (Gottman et al., 1998; Williams, 2007; Niven et al., 2012; Bloch et al., 2014; Levenson et al., 2014). Importantly, the present findings build upon those previous results by demonstrating related benefits emerging between people and brands when antecedent-focused IER is employed.

The current findings also have implications for understanding people’s connections to brands. Prior research has emphasized the nature of the relationship between customers and brands (Fournier, 1998) and highlighted the important role of customer emotions in forging bonds with brands (Jacoby and Chestnut, 1978; Dick and Basu, 1994; Oliver, 1999; Thomson et al., 2005; Grisaffe and Nguyen, 2011; Iglesias et al., 2011). We build on this tradition by demonstrating that when frontline employees engage in antecedent-focused rather than response-focused IER to manage their customers’ emotions, the brands they represent experience increased brand trust and brand loyalty driven by changes in the customers’ emotions. Such benefits may be especially important following brand failures, as consumers often experience negative emotions following such failures (Smith et al., 1999; Aaker et al., 2004), and these emotions can in turn precede negative customer actions, such as spreading negative word-of-mouth or engaging third parties (Smith and Bolton, 2002; Bonifield and Cole, 2007; Kalamas et al., 2008). By addressing customers’ emotions using antecedent-focused emotion regulation, brands can mitigate the negative fallout from such failures and insulate their relationship with customers from the damaging effects of such failures.

Our research demonstrates the brand benefits of antecedent-focused IER, producing consistent findings across five experiments. We especially focus on the benefits of antecedent-focused emotion regulation as opposed to the detriments of response-focused emotion regulation given the findings of Study 2, which found that antecedent-focused regulation resulted in improved customer emotions and brand perceptions compared to the control condition in which IER was not attempted. The benefits of antecedent-focused IER compared to response-focused IER were consistently observed regardless of whether customers’ emotions were driven by a product failure (Studies 1 and 3), a service failure (Studies 2 and 5), or personal concerns unrelated to the brand (Study 4). Compared to response-focused IER, antecedent-focused IER generally resulted in changes in both positive (happiness) and negative (anger) emotions, which mediated its influence on brand loyalty and trust. However, the genesis of customers’ emotions may somewhat alter the pathway by which IER influences brand loyalty and brand trust. We demonstrate that the causal pathway we establish involving anger is highly consistent across all four of the studies examining failures on the part of the brand, even when the benefits of the customer service interaction are made explicit to customers. Following product or service failures, anger is often the dominant customer response and has important consequences which shape their reaction to the failure (Smith et al., 1999; Smith and Bolton, 2002; Aaker et al., 2004; Bonifield and Cole, 2007; Kalamas et al., 2008). Thus, it makes sense that changes in customer anger would be central to resulting brand benefits arising due to differences in IER strategy. However, when we examined IER directed at customer emotions that were unrelated to the brand, we found that changes in customer happiness were central to the effects on brand trust and loyalty. This difference likely reflects the fact that customers were feeling less anger in this context, and thus changes in happiness following IER were essential to producing the effect. Happiness may also reflect increased gratitude or surprise that the employee sought to improve their emotional state despite it arising for reasons unrelated to the brand they represent. Prior research has demonstrated that actions by employees that exceed customers’ expectations often result in greater satisfaction and other improved outcomes (Menon and Dubé, 2000), consistent with the differences in customer reactions we observe in this study. Future research can further examine how the genesis of customers’ emotions may moderate the present findings. For example, it could be the case that when customers’ emotions arise due to anxieties about a purchase they are considering (Luce, 1998; Luce et al., 2001), such as anxious parents shopping for a car seat, changes in customer happiness in response to employee IER may also play a central role in producing brand benefits.

While response-focused IER generally resulted in worse outcomes for brands than antecedent-focused IER, we demonstrated an important boundary condition of this effect. When the IER attempt is made visible and customers are explicitly informed that the employee seeks to improve their emotional state, both the response-focused and antecedent-focused IER result in similarly positive outcomes. This finding is somewhat distinct from findings from the social support literature, in which making interpersonal social support visible undermines its positive benefits (Bolger et al., 2000; Bolger and Amarel, 2007). These discrepant findings may be due to two key differences between the types of interactions we examine here and previous research. First, prior research has established that visible support results in diminished benefits because its provision implies that the recipient of support is incapable of meeting the challenge posed to them (Bolger and Amarel, 2007). In the current context, it seems unlikely that the provision of support would signal that employees believe customers are unable to manage their own emotions. Rather, attempting to help the customer regulate their emotions may be viewed as reasonable and expected in this customer service context. Second, visible support has often been examined in relationships that are clearly communal, with both parties caring and valuing one another’s outcomes (Clark and Mills, 1993; Bolger et al., 2000). In such relationships, it is assumed that the person providing support has benevolent intentions toward the target of support. In the contexts investigated here, it may not be clear to customers whether the relationship is communal or exchange-based (Clark and Mills, 1979; Clark et al., 1987). Therefore, when the IER attempt is made visible, it signals to the customer that the employee and brand have benevolent intentions toward them and the relationship is more likely to be communal. We demonstrate that the benefits of making the IER attempt visible are mediated by changes in judgments of the benevolence of the employee, consistent with the notion that visible IER makes the relationship appear to be more communal.

The present research represents an important advance in understanding the emotional dynamics underlying interactions between employees and customers, particularly in customer service contexts. Prior research has focused on how each party manages their emotions independently. For example, research on emotional labor has established that employees who use antecedent-focused emotion regulation compared to response-focused emotion regulation to manage their own emotional reactions typically experience more job satisfaction, less burnout, and better performance (Côté and Morgan, 2002; Groth et al., 2009; Judge et al., 2009; Hülsheger et al., 2010; Chi et al., 2011; Hülsheger and Schewe, 2011; Kammeyer-Mueller et al., 2013; Grandey and Gabriel, 2015). Alternately, research has examined how customers manage their own emotions in response to brand failures (Strizhakova et al., 2012; Sengupta et al., 2015; Balaji et al., 2017). The present research advances past work considering each party’s emotions in isolation by examining how one party may intervene to influence the other party’s emotional state. Future research may extend these findings by examining interactions between each party’s strategy for managing their own emotions and the IER strategy implemented by the employee. It may be the case, for example, that an employee’s strategy for managing their own emotions interacts with the strategy they implement to manage the customer’s emotions, such that the best outcomes occur when antecedent-focused strategies are employed for both.

A general limitation of the present research is that all of the studies instructed participants to imagine an interaction with a salesperson or service representative. This hypothetical, scenario method may result in less consumer involvement, and thus milder emotional reactions, than a real brand encounter (Hess et al., 2003). However, this approach had the advantage of allowing us to control important aspects of the interaction, including the specific manner in which IER was implemented. Additionally, the present studies all focused on one-time interactions between a consumer and an employee rather than analyzing ongoing relationships. Previous research has demonstrated that consumers can form loyalty relationships with specific employees in consumer settings (Reynolds and Arnold, 2000), and responses to IER attempts may vary based on the previous relationship between a customer and an employee. This possibility presents an interesting avenue for future research. The present research also avoided using real brands to demonstrate the effects of IER independent from consumers’ prior experiences with the brand. However, future work could examine how IER interacts with key brand features. For example, prior research demonstrates that people respond differently to brand failures based on the brand’s personality (Aaker et al., 2004). Similarly, the efficacy of IER strategies may interact with brand personalities. For instance, authentic brands may especially benefit from deploying antecedent-focused IER to address customers’ emotions. Additionally, the present research only examined these IER processes within North American culture. Different processes and effects may emerge in diverse cultures, especially those that differ in interdependence and norms for emotional experience and expression. Future research should consider examining other business contexts and cultures.

The current research has several important managerial implications. For example, the present findings could be utilized to shape employee training in customer relationship management and complaint handling. Specifically, our findings show that frontline employees should focus on not only regulating their own emotions but also helping customers manage their feelings by using antecedent-focused IER. Our research demonstrates that even when the customer service agent successfully resolves the problem that gave rise to customers’ negative emotions, using a response-focused IER strategy still resulted in worse customer emotional reactions and detrimental outcomes for the brand. By deploying antecedent-focused IER, customer service representatives can not only improve customers’ emotional states but also enhance their brand loyalty and brand trust. The negative emotions customers experience in response to a brand failure can have numerous negative consequences, including damaging the brand relationship and leading to more negative word-of-mouth (Fournier, 1998; Kalamas et al., 2008). By addressing consumers’ emotional states, employees can help insulate the brand relationship from negative consequences following product or service failures. Additionally, the present research indicates that IER can also be used by frontline employees as a mechanism to strengthen brand trust and loyalty even in cases in which no service or product problem exists. For low reactance customers, helping them manage negative feelings arising for reasons unrelated to the brand can result in improved brand loyalty and trust.

Unfortunately, frontline employees frequently select response-focused IER in highly intense negative situations (Menon and Dubé, 2000, 2004; Little et al., 2013). In addition to training employees to instead deploy antecedent-focused IER, firms can also recover from the selection of response-focused IER by making the IER attempt visible to customers. By clearly communicating that the employee cares about the customer’s emotional state and values improving their emotions, employees can recover from initially engaging in a less advantageous IER strategy. The present findings may also provide insights that can be used during the hiring process to identify prospective candidates that might be well-suited to customer service. For example, firms might prioritize hiring prospective employees who are more likely to habitually use antecedent-focused IER in their daily lives.

Customers can experience a suite of emotions during the course of doing business with firms, from anxieties sparked by emotional purchases to anger generated by brand failures. The present research clarifies how the strategies employees use to manage customers’ emotions may have consequences not only for customers’ emotional states but also for their relationship with the brand. We demonstrate that, compared to response-focused IER, antecedent-focused IER results in enhanced brand loyalty and trust, due to concordant changes in customers’ emotions. The present findings build on prior theories that emphasize the role of emotional connections in shaping customers’ brand loyalty (Jacoby and Chestnut, 1978; Fournier, 1998; Evanschitzky et al., 2006; Grisaffe and Nguyen, 2011), demonstrating that successfully managing negative customer emotions can result in improved brand trust and brand loyalty. There are also parallels to prior demonstrations that IER between people results in increased trust and relationship satisfaction (Niven et al., 2007, 2012; Williams, 2007; Bloch et al., 2014). In light of the present findings, firms may seek to train their employees to utilize antecedent-focused IER during interactions with customers. Doing so should not only improve customers’ emotional states but also their brand loyalty and brand trust.

## Data Availability Statement

The raw data supporting the conclusions of this article will be made available by the authors, without undue reservation.

## Ethics Statement

The studies involving human participants were reviewed and approved by Institutional Review Board, Temple University. Written informed consent for participation was not required for this study in accordance with the national legislation and the institutional requirements.

## Author Contributions

CR and NO designed the experiments, collected and analyzed the data, and wrote the manuscript. Both authors contributed to the article and approved the submitted version.

## Conflict of Interest

The authors declare that the research was conducted in the absence of any commercial or financial relationships that could be construed as a potential conflict of interest.

## Publisher’s Note

All claims expressed in this article are solely those of the authors and do not necessarily represent those of their affiliated organizations, or those of the publisher, the editors and the reviewers. Any product that may be evaluated in this article, or claim that may be made by its manufacturer, is not guaranteed or endorsed by the publisher.
