# Supplementary material for: Interpersonal Emotion Regulation: Consequences for Brands in Customer Service Interactions
Source: Front Psychol. 2022 Jun 9;13:872670. doi: 10.3389/fpsyg.2022.872670 (PMC9223079; doi:10.3389/fpsyg.2022.872670)
Supplement: Supplementary file 1 [file Data_Sheet_1.PDF]

Interpersonal Emotion Regulation: Consequences for Brands in Customer Service Interactions

Supplemental Materials

Supplemental Table 1

Demographics for each experiment.

Study	Sample	Mean Age	Mean Income	% Female	% White/ Caucasian	% African/ African American	% Asian/ Asian American
1	255	19.95	$28,729	38.8	76.5	1.2	4.0
2	308	20.5	$11,847	45.7	71.4	7.1	18.8
3	351	34.8	$42,938	39.3	75.2	13.4	9.7
4	198	34.1	$45,846	37.9	81.8	10.6	4.5
5	498	35.2	$48,198	51.0	75.0	11.4	5.7

Study 1 Emotion Regulation Manipulation

"A few weeks ago, you bought a new laptop. You are using your new laptop to complete a project that is due tomorrow. Suddenly, the laptop displays an error message and turns off. You try several times, but you can not turn your laptop on again. This is very frustrating since your project is due tomorrow, so you need this problem fixed right away. You call the customer support number for your laptop and are connected with a representative. You explain the situation and let them know how upset you are, since this is a new computer and should be working properly. The customer service representative tells you, "I'm sorry for the problem, and I will be able to fix it. [It's good that you called in, since we can also make sure your virus protection software is running properly] (Antecedent-focused) [Please take a deep breath and calm down"] (Response-focused).

Study 1 Measures

Feelings about service interaction

Thinking about your interaction with the customer service representative, to what extent do you feel: Happy, Angry, Frustrated, Irritable, Hostile, Pleased, Joyful (1= Not at all, 7=Extremely)

Evaluation of service interaction

How would you rate your interaction with the customer service representative? (1= Extremely negative, 7=Extremely positive)

Brand loyalty

Select the option that is relevant to you about the laptop brand. (1= Strongly disagree, 7=Strongly agree)

I would say positive things about this brand to other people

When I need another laptop, I would consider this brand

I would encourage friends and relatives to buy this brand

I would not consider switching away from this brand

As long as the present service continues, I doubt that I would switch to another brand

Brand trust (1= Strongly disagree, 7=Strongly agree)

Select the option that is relevant to you about the laptop brand.

This brand meets my expectations

I feel confidence in this brand

This brand never disappoints me

This brand would be honest and sincere in addressing my concerns

I could rely on this brand to solve the problem

This brand would make any effort to satisfy me

This brand would compensate me in some way for the problem with the services

Supplemental Figure 1

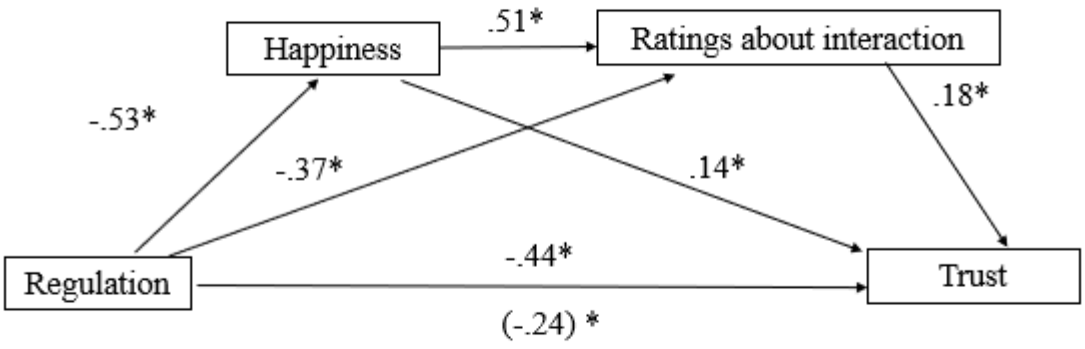

Interpersonal emotion regulation increases trust through changing emotional responses towards the service interaction. * *p*<.05 (Value in parentheses indicates the effect of emotion regulation on the dependent variable after controlling for the mediators)

Study 2 Emotion Regulation Manipulation

"You recently received an official letter from your bank stating that you are charged an overdraft fee on your account. The letter claims your account is overdrawn for three days, but you are sure that you did not make any transactions that would have caused your account balance to fall below $0. This is very frustrating and you are anxious to avoid any penalties on your account. You call the bank's customer service and are connected with a representative. You explain the situation and let them know how upset you are. The customer service representative tells you, "I'm sorry for the problem, and I will be able to fix it.(Control) [It's good that you called in, since we can also make sure all the other information is correct on your account today] (Antecedent-focused) [Please take a deep breath and calm down]" (Response focused). After a few minutes, the representative solves the issue and you do not incur any penalties."

Study 2 Measures Added

How would you rate the customer service representative? (1= Not at all, 7=Extremely)

Warm, Friendly, Competent, Capable

Supplemental Figure 2

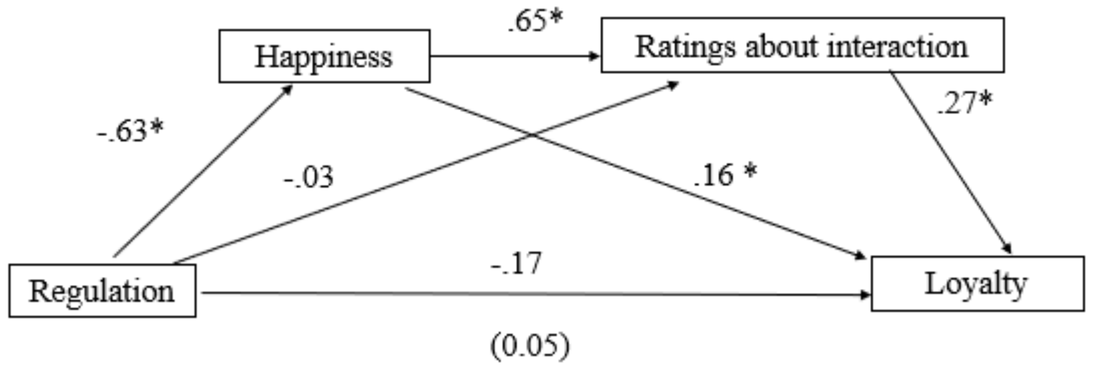

Interpersonal emotion regulation increases loyalty through changing emotional responses towards the service interaction. * $p<.05$ Value in parentheses indicates the effect of emotion regulation on the dependent variable after controlling for the mediators.

Supplemental Figure 3

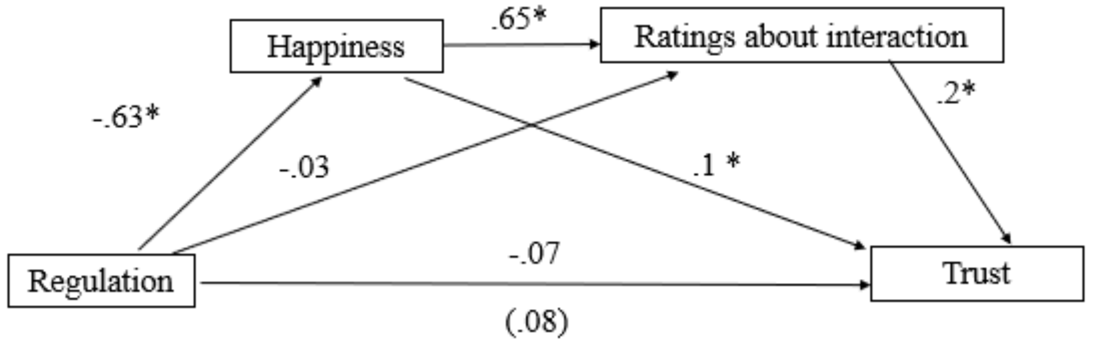

Interpersonal emotion regulation increases trust through changing emotional responses towards the service interaction. * *p*<.05. Value in parentheses indicates the effect of emotion regulation on the dependent variable after controlling for the mediators.

Supplemental Figure 4

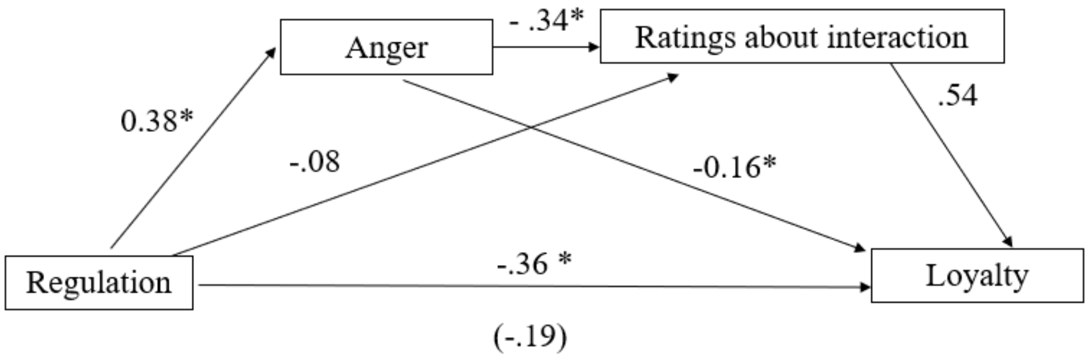

Interpersonal emotion regulation increases loyalty through changing emotional responses towards the service interaction. * *p*<.05 Value in parentheses indicates the effect of emotion regulation on the dependent variable after controlling for the mediators.

Study 3 Emotion Regulation Manipulation

"A few weeks ago, you bought a new laptop. You are using your new laptop to complete a project that is due tomorrow. Suddenly, the laptop displays an error message and turns off. You try several times, but you can not turn your laptop on again. This is very frustrating since your project is due tomorrow, so you need this problem fixed right away.

You call the customer support number for your laptop and are connected with a representative. You explain the situation and let them know how upset you are, since this is a new computer and should be working properly. The customer service representative tells you, "I'm sorry for the problem, and I will be able to fix it. [It's good that you called in, since we can

also make sure your virus protection software is running properly] (Antecedent-focused) [Please take a deep breath and calm down] (Response-focused)."

After diagnosing the problem in your laptop, the representative solves the problem. While solving the main problem, the representative also confirms your virus protection software is up to date.

Study 4 Emotion Regulation Manipulation

"Imagine that you were on your way to meet with friends, but traffic was awful, and you couldn't make it to meet them. Your friends are very upset with you for not meeting them, which upsets you as well. On the way home, you stop by a clothing store to pick up a shirt. You're having trouble finding the one you want in your size when a salesperson offers to help you. While the salesperson is searching for the right size, the salesperson asks about your day. You share what happened and how upset you feel. The salesperson says, "I am sorry to hear that. In these situations, it sometimes helps me [to remember it's just one bad day and overall my friends care about me] (Antecedent-focused) [to calm myself down by taking long, deep breaths and trying to smile more.] (Response-focused)" The salesperson then finds the shirt you wanted."

Study 4 Added Measures

I considered the salesperson's advice to be an intrusion

I felt like doing the opposite of the salesperson's advice

I tried not to let the salesperson's advice influence me

It irritated me when the salesperson suggested things that I should do

I felt upset that the salesperson tried to influence the way I feel

(1= Strongly disagree, 7=Strongly agree)

Study 5 Visibility Manipulation

In visible conditions the sentence below was included in the beginning of IER manipulation:

"I can tell you are upset, and part of my job is to help you feel better."
